# Clinical characteristics and risk factor analysis of multidrug-resistant bacterial bloodstream infections in adult acute leukemia patients

**DOI:** 10.3389/fmicb.2026.1850927

**Published:** 2026-06-04

**Authors:** Xiaodan Wang, Shuiqing He, Weigong Chen, Ying Li, Jingya Bu

**Affiliations:** 1 Department of Clinical Laboratory, Second Affiliated Hospital of Dalian Medical University, Dalian, China; 2 Department of Rheumatology, Second Affiliated Hospital of Dalian Medical University, Dalian, China; 3 Department of Laboratory of Genetic Testing, Dalian Innovation Institute of Stem Cell and Precision Medicine, Dalian, China

**Keywords:** acute leukemia, bloodstream infections, hypoproteinemia, MDR, risk factor

## Abstract

**Background:**

Bloodstream infection (BSI) is a common complication in adults with acute leukemia (AL), and the problem of multidrug-resistant (MDR) bacterial infection is exacerbated by inappropriate antibiotic use; therefore, identifying its clinical features and risk factors is critical to improving clinical outcomes.

**Methods:**

LASSO regression was employed for variable selection, followed by multivariate logistic regression to identify independent risk factors for multidrug-resistant bacterial bloodstream infection. Given the limited number of 30-day mortality events, LASSO regression combined with univariate logistic regression was used to preliminarily explore factors associated with 30-day mortality. Internal validation was performed using the bootstrap method with 1,000 resamples.

**Results:**

The median age of the patients was 56 years. The 30-day all-cause mortality rate was 10.5%. Gram-negative bacteria accounted for 71.053% of the infections, with *Escherichia coli (E. coli)* and *Klebsiella pneumoniae (K. pneumoniae)* being most common. Among the 105 identified MDR isolates (55.263%), both Gram-negative and Gram-positive bacteria exhibited high resistance rates to fluoroquinolones and penicillins. Invasive procedures and hypoproteinemia were identified as independent risk factors for MDR bacterial infection. Septic shock, CRP, and the AST/ALT ratio may be associated with an increased risk of 30-day mortality, whereas length of hospitalization may serve as a protective factor. Given the limited number of events, the study may have been underpowered to detect a definitive survival impact of MDR infection, warranting validation in larger cohorts.

**Conclusion:**

This study provides important epidemiological data on blood infections in patients with AL in northeastern China, and provides a reference basis for optimizing infection prevention and control strategies based on local pathogens.

## 1 Introduction

Acute leukemia is a common hematopoietic malignancy that includes acute myeloid leukemia (AML) and acute lymphoblastic leukemia (ALL). Immunosuppression often develops after chemotherapy, making bloodstream infections (BSI) more common in patients with hematologic malignancies (Cui et al., 2025; Chorão et al., 2026; Baccelli et al., 2025). Studies have reported that the incidence of BSI in patients with AL can reach about 30%, with a steadily increasing detection rate of MDR bacteria (Baccelli et al., 2025). Delayed or restricted effective therapy for infections caused by MDR bacteria increases the risk of death (Ruhnke et al., 2014).

In recent years, with the empirical use of antibiotics, the challenges posed by drug-resistant bacteria in clinical treatment have become increasingly prominent (Khateb et al., 2025; Merdad et al., 2023), and the prevention of infection has become an important step in the diagnosis and treatment of patients with blood cancer. The main pathogens of BSIs in patients with acute leukemia are Gram-negative bacilli and Gram-positive cocci, with Gram-negative bacteria having a higher infection rate.

Previous studies have identified multiple risk factors for MDR bacteria bloodstream infections, including neutropenia, invasive procedures, and previous antibiotic exposure. Neutropenia and invasive procedures increase the risk of infection (Trecarichi et al., 2023). Neutropenia is a common adverse reaction after chemotherapy, which significantly increases the risk of invasive infection and exacerbates the risk of death (Chen et al., 2025). In the bloodstream infections of patients with acute leukemia caused by multiple pathogens, hypoproteinemia is often present as an independent risk factor (Cui et al., 2025; Jeddi et al., 2011). Hypoproteinemia reflects abnormalities in the patient’s nutritional status, immune system, and metabolic function, which may create a more favorable environment for multidrug-resistant bacterial infections.

However, data on MDR bacterial BSIs in patients with acute leukemia are limited, this study aimed to analyze the clinical characteristics of MDR bacterial BSIs and related high-risk factors and provide a reference for clinical empirical antibiotic use and prevention of BSIs in patients with acute leukemia.

## 2 Materials and methods

### 2.1 Research subjects and groups

This study is a retrospective study, and patients admitted to the hematology ward of the Second Affiliated Hospital of Dalian Medical University from January 2019 to December 2025 who met the following inclusion and exclusion criteria were included in this study. Inclusion criteria: 1. Diagnosed with acute leukemia. 2. Age > 18 years. 3. Blood culture is positive, with or without septic shock, and pathogenic bacteria are detected and drug susceptibility results are present. 4. Clinical case information and experimental data are complete. Exclusion criteria: 1. Patients with non-acute leukemia. 2. Fungal infection. 3. Incomplete clinical case information. 4. Considering contaminating bacteria. According to the drug susceptibility results, 190 patients were divided into 105 in the multidrug-resistant bacterial infection group (MDR) and 85 in the non-multidrug-resistant infection group (NMDR).

### 2.2 Data collection

This study was collected through the electronic information system of the Second Affiliated Hospital of Dalian Medical University. The specific information is as follows: age, gender, disease diagnosis, length of hospitalization, presence of underlying diseases, presence of invasive procedures, transplantation, chemotherapy, complications, empiric antibiotic use, death (30 days after BSI), whether it was a nosocomial acquired infection, time of positive blood culture, and corresponding laboratory test results. Laboratory parameters were measured within 24 h of blood culture collection.

### 2.3 Definitions

Bloodstream infection (BSI) is defined as the presence of fever or other clinical manifestations suggestive of systemic in fection in AL patients, accompanied by at least one positive blood culture obtained from a peripheral venous sample, with specimen contamination ruled out. For common commensals (e.g., coagulase-negative staphylococci), at least two separate blood culture sets were required for diagnosis.

Multidrug resistance refers to antimicrobial resistance to ≥ 3 categories of different mechanisms of action.

Neutropenia refers to the absolute value of neutrophils < 0.5 × 10^9 /L.

Duration of neutropenia refers to the number of days from the first drop of absolute neutrophil count (ANC) to ≤ 0.5 × 10^9/L until two consecutive ANC measurements recover to ≥ 0.5 × 10^9/L.

Appropriate empiric antibiotic therapy refers to the use of at least one antibiotic that is sensitive to the susceptibility result before the drug susceptibility result is shown; If not, it is defined as inappropriate empiric antibiotic therapy.

It is generally believed that hypoproteinemia is hypoproteinemia when the total plasma protein is < 60 g/L or the albumin < 35 g/L.

Invasive procedures are defined as the presence of any indwelling invasive device at the time of bloodstream infection (BSI) diagnosis, including peripherally inserted central catheters (PICC), central venous catheters (CVC), or urinary catheters.

MCV, Mean Corpuscular Volume, AST, Aspartate Aminotransferase, ALT, Alanine Aminotransferase, GLB, Globulin, and CRP, C-reactive Protein.

### 2.4 Statistical analysis

Categorical variables are expressed in frequency, and chi-square test or Fisher’s exact test is used for comparison between groups. Continuous variables that conform to normal distribution are expressed as mean ± standard deviation (SD), while non-normally distributed data are expressed as median and interquartile ranges [M (Q1, Q3)], and between groups comparisons are compared using the *t*-test for normally distributed variables and the Mann–Whitney U test for non-normally distributed variables. Variable selection was performed using LASSO regression, and the selected variables were then included in a multivariate logistic regression model to identify independent risk factors for bloodstream infection of multidrug-resistant bacteria, internal validation was performed using the bootstrap method with 1,000 resamples. Considering the small sample size for 30-day mortality, we first performed LASSO regression for variable selection, followed by univariate logistic regression analysis on the selected variables, to preliminarily evaluate factors associated with 30-day mortality risk in adult patients with acute leukemia. *P* < 0.05 was considered statistically significant. Data statistical analysis and visualization were accomplished using R (version 4.2.1) and GraphPad Prism (version 9.5.1) software.

## 3 Results

### 3.1 Patient population

A total of 190 patients with acute leukemia who developed bloodstream infection during hospitalization were included in this study, including 120 (63.158%) male patients and 70 (36.842%) female patients. The median age of patients was 56 years (interquartile range 44–64 years). 146 patients (76.842%) had acute myeloid leukemia and 44 (23.158%) had acute lymphoblastic leukemia. 21 patients (10.053%) received hematopoietic stem cell transplantation and 181 patients (95.263%) received chemotherapy. In this study, all patients had fever symptoms during bloodstream infection, including 66 cases (34.737%) with pulmonary infection, 43 cases (22.632%) with intestinal infection, 36 cases (18.943%) with oral infection, 28 cases (14.737%) with septic shock, 27 cases (14.211%) with perianal abscess infection, 21 cases (11.053%) with skin infection, 15 cases (7.895%) with urinary tract infection, and 2 cases (1.053%) with genital tract infection. Other patient characteristics are detailed in Table 1. At the same time, the number of MDR isolates, the number of positive blood cultures and the prevalence of MDR were statistically analyzed by year, as shown in Supplementary Figure S1 and Supplementary Table S1 for details.

**TABLE 1:** Participant information and baseline characteristics.

Characteristic	All patients (*n* = 190)	MDR (*n* = 105)	NMDR (*n* = 85)	*P*-value
Gender, n (%)				
Male	120 (63.158)	71 (67.619)	49 (57.647)	0.1565
Female	70 (36.842)	34 (32.381)	36 (42.353)	
Age, median (IQR)	56 (46,64)	55 (42,61.5)	59 (51.5,69)	0.0019
Diagnosis, n (%)				
AML	146 (76.842)	74 (70.476)	72 (84.706)	0.0208
ALL	44 (23.158)	31 (29.524)	13 (15.294)	
Length of hospital stay, d, median (IQR)				
	24 (15, 31)	26 (21, 34)	22 (9, 28)	0.0003
Underlying medical conditions, n (%)				
Diabetes	28 (14.737)	13 (12.381)	15 (17.647)	0.3422
Hypertension	37 (19.474)	22 (20.952)	15 (17.647)	0.5164
No underlying medical conditions, n (%)				
	105 (55.263)	63 (60.000)	42 (52.941)	0.1444
Invasive procedure, n (%)				
Urinary catheter	8 (4.211)	4 (3.810)	4 (4.706)	0.7597
PICC/CVC	97 (51.053)	68 (64.762)	29 (34.118)	< 0.001
Hematopoietic stem cell transplantation, n (%)				
	21 (11.053)	13 (12.381)	8 (9.412)	0.5163
Chemotherapy, n (%)	181 (95.263)	101 (96.190)	80 (94.118)	0.5036
Complications, n (%)				
Septic shock	28 (14.737)	16 (15.238)	12 (14.118)	0.8285
Oral infection	36 (18.943)	13 (12.381)	23 (27.059)	0.0103
Pulmonary infection	66 (34.737)	32 (30.476)	34 (40.000)	0.1704
Intestinal infection	43 (22.632)	28 (26.667)	15 (17.647)	0.1396
Perianal abscess infection	27 (14.211)	17 (16.190)	10 (11.765)	0.3850
Urinary tract infection	15 (7.895)	9 (8.571)	6 (7.059)	0.7006
Genital tract infection	2 (1.053)	0 (0.000)	2 (2.353)	0.1141
Skin infection	21 (11.053)	11 (10.476)	10 (11.765)	0.7782
Death, n (%)	20 (10.526)	10 (9.524)	10 (11.765)	0.6168
Hospital-acquired infection, n (%)				
	132 (69.474)	79 (75.238)	53 (62.353)	0.0552
Inappropriate empiric antibiotic treatment, n (%)				
	55 (28.947)	33 (31.429)	22 (25.882)	0.4019
Neutropenia, n (%)	154 (81.053)	87 (82.857)	67 (78.824)	0.4805
Duration of neutropenia, d, median (IQR)	14 (8, 20.5)	16 (10, 21)	10 (6, 18)	0.0245
Microscopic examination results, n (%)				
Gram-negative bacilli	135 (71.053)	75 (71.429)	60 (70.588)	0.8989
Gram-positive cocci	55 (28.947)	30 (28.571)	25 (29.412)	
Time to positive blood culture, h, median (IQR)				
	12.2 (10.04, 18)	12.10 (10, 17.78)	12.60 (10.09, 18)	0.6613

### 3.2 Microbiology and antimicrobial susceptibility of the isolates

**FIGURE 1:**
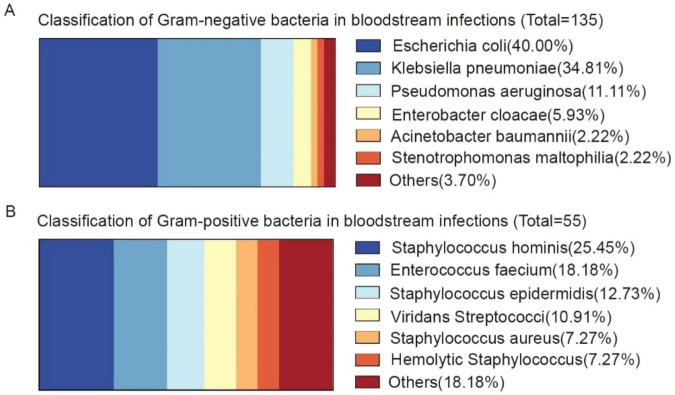
Blood culture positive bacterial classification chart. **(A)** Classification of Gram-negative bacteria in bloodstream infections. **(B)** Classification of Gram-positive bacteria in bloodstream infections.

Among 190 patients with bloodstream infections, there were 135 cases of Gram-negative bacillus bloodstream infections (71.053%), and 55 cases of Gram-positive cocci infections (28.947%), the specific bacterial species and their proportions are shown in Figure 1.

Among the multidrug-resistant Gram-negative bacilli, *E. coli* was isolated in 39 cases (52.000%), followed by *K. pneumoniae* in 25 cases (33.333%). Among the multidrug-resistant Gram-positive cocci, *Enterococcus faecium (E. faecium)* was isolated in 10 cases (33.333%), followed by *Staphylococcus hominis (S. hominis)* in 9 cases (30.000%).

A total of 105 cases of multidrug-resistant bacteria were identified, including 75 cases of Gram-negative bacteria (71.429%) and 30 cases of Gram-positive cocci (28.571%). Further analysis of their drug resistance profiles was conducted separately. Nearly all multidrug-resistant Gram-negative bacilli were resistant to fluoroquinolones (ciprofloxacin and levofloxacin), with a resistance rate of 97.333% (73/75) to ciprofloxacin and 84.000% (63/75) to levofloxacin. Trimethoprim/Sulfamethox and Piperacillin also exhibit high resistance rates. In addition, cephalosporins demonstrate relatively limited efficacy against multidrug-resistant Gram-negative bacilli. Details are provided in Table 2.

**TABLE 2:** Analysis of drug resistance rate of Gram-negative bacteria in multidrug-resistant bacteria, n (%).

Microorganisms	Total	*Escherichia coli*	*Klebsiella pneumoniae*	*Enterobacter cloacae*	*Acinetobacter baumannii*	Others
Total	75	39 (52.000)	25 (33.333)	7 (9.333)	2 (2.667)	2 (2.667)
Ciprofloxacin	73	39 (53.425)	23 (31.507)	7 (9.589)	2 (2.740)	2 (2.740)
Levofloxacin	63	39 (61.905)	17 (26.984)	5 (7.937)	0 (0.000)	2 (3.175)
Ampicillin/Sulbactam	53	32 (60.377)	20 (37.736)	NA	0 (0.000)	1 (1.887)
Piperacillin	67	33 (49.254)	24 (35.821)	7 (10.448)	2 (2.985)	1 (1.493)
Piperacillin/Tazobactam	35	16 (45.714)	13 (37.143)	4 (11.429)	2 (5.714)	0 (0.000)
Cefazolin	59	34 (57.627)	23 (38.983)	NA	NA	2 (3.390)
Cefuroxime (sodium)	62	32 (51.613)	22 (35.484)	6 (9.677)	NA	2 (3.226)
Cefuroxime (ester)	63	32 (50.794)	23 (36.508)	6 (9.524)	NA	2 (3.175)
Ceftazidime	47	24 (51.064)	15 (31.915)	7 (14.894)	0 (0.000)	1 (2.128)
Ceftriaxone	62	31 (50.000)	21 (33.871)	7 (11.290)	1 (1.613)	2 (3.226)
Cefepime	43	21 (48.837)	13 (30.233)	6 (13.953)	2 (4.651)	1 (2.326)
Aztreonam	46	23 (50.000)	17 (36.957)	5 (10.870)	NA	1 (2.174)
Imipenem	29	11 (37.931)	11 (37.931)	4 (13.793)	2 (6.897)	1 (3.448)
Meropenem	30	11 (36.667)	12 (40.000)	4 (13.333)	2 (6.667)	1 (3.333)
Cefoperazone/Sulbactam	24	11 (45.833)	11 (45.833)	2 (8.333)	0 (0.000)	0 (0.000)
Gentamicin	44	26 (59.091)	13 (29.545)	2 (4.545)	2 (4.545)	1 (2.273)
Trimethoprim/Sulfamethox	73	39 (53.425)	23 (31.507)	7 (9.589)	2 (2.740)	2 (2.740)

Similarly, almost all multidrug-resistant Gram-positive cocci were resistant to penicillin, with a resistance rate of 96.667% (29/30), followed by erythromycin at 93.333% (28/30). Resistance to fluoroquinolones remained relatively high, with a resistance rate of 83.333% (25/30) to levofloxacin and 73.333% (22/30) to ciprofloxacin. Details are presented in Table 3.

**TABLE 3:** Analysis of drug resistance rate of Gram-positive bacteria in multidrug-resistant bacteria, n (%).

Microorganisms	Total	*Enterococcus faecium*	*Staphylococcus hominis*	*Hemolytic Staphylococcus*	*Staphylococcus epidermidis*	Others
Total	30	10 (33.333)	9 (30.000)	4 (13.333)	3 (10.000)	4 (13.333)
Penicillin	29	9 (31.034)	9 (31.034)	4 (13.793)	3 (10.345)	4 (13.793)
Erythromycin	28	10 (35.714)	8 (28.571)	3 (10.714)	3 (10.714)	4 (14.286)
Levofloxacin	25	8 (32.000)	7 (28.000)	3 (12.000)	3 (12.000)	4 (16.000)
Ciprofloxacin	22	8 (36.364)	7 (31.818)	3 (13.636)	2 (9.091)	2 (9.091)
Tetracycline	19	6 (31.579)	5 (26.316)	3 (15.789)	2 (10.526)	3 (15.789)
Oxacillin	17	NA	8 (47.059)	4 (23.529)	2 (11.765)	3 (17.647)
Clindamycin	15	NA	9 (60.000)	1 (6.667)	2 (13.333)	3 (20.000)
Moxifloxacin	12	NA	4 (33.333)	4 (33.333)	1 (8.333)	3 (25.000)

### 3.3 Risk factors for multidrug-resistant bacteria with bloodstream infection

**FIGURE 2:**
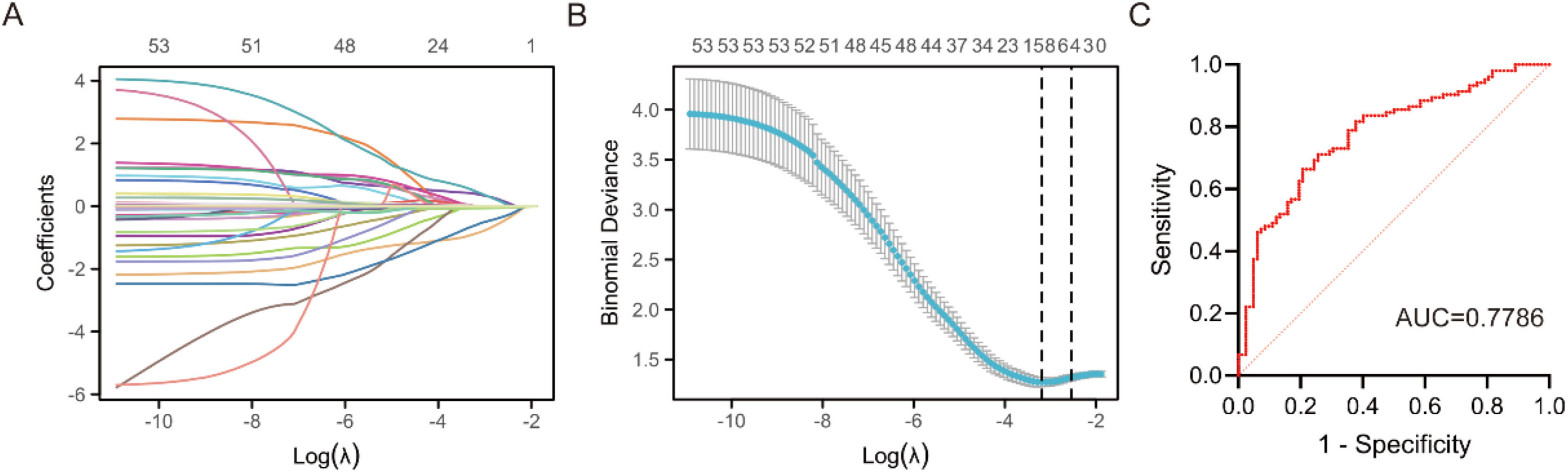
Risk factors for multidrug-resistant bacterial bloodstream infection in adult patients with acute leukemia screened by Lasso regression, and the ROC curve of the corresponding multivariate logistic regression model. **(A)** Plot of the LASSO coefficients. The horizontal axis represents the logarithmic value of lambda [log(λ)], and the vertical axis represents the coefficient values of the variables. **(B)** Cross-validation error curve. Bottom *x*-axis: represents the logarithm of lambda [log(λ)]. Numbers on the top *x*-axis: represent the number of non-zero coefficient variables corresponding to each lambda value. *y*-axis: represents the model’s -2 times the log-likelihood function value (deviance) under different metrics **(C)** ROC curve of the risk factor model for multidrug-resistant bacterial bloodstream infection.

Through LASSO regression, it was identified that invasive procedures, oral infections, MCV, AST/ALT ratio, hypoproteinemia, and GLB might be potential risk factors for multidrug-resistant bacterial bloodstream infection in adult patients with acute leukemia. These variables were included in a multivariate logistic regression model for construction. The results showed that invasive procedures (OR = 3.533, 95% CI: 1.795–7.149, *p* = 0.0003) and hypoproteinemia (OR = 3.123, 95% CI: 1.296–7.905, *p* = 0.0129) were significant independent risk factors for multidrug-resistant bacterial bloodstream infection in adult patients with acute leukemia. The multivariate regression model achieved an AUC of 0.7786 (95% CI: 0.7121–0.8452), and the Hosmer–Lemeshow goodness-of-fit test yielded a *p*-value of 0.7676, indicating good model fit, as detailed in Figure 2 and Table 4. The constructed multivariate logistic regression model was internally validated using the bootstrap method with 1,000 resamples. The results showed a mean bias-corrected AUC of 0.769 (95% CI: 0.751–0.779), confirming that the model exhibited good stability and no significant overfitting.

**TABLE 4:** Multivariate analysis of risk factors for bloodstream infection of multidrug-resistant bacteria.

Variable	MDR (*n* = 105)	NMDR (*n* = 85)	OR	95% CI	*P*-value
Invasive procedure, n (%)	72 (68.571)	33 (38.824)	3.533	1.795–7.149	0.0003
Oral infection, n (%)	13 (12.381)	23 (27.059)	0.3450	0.1433–0.7983	0.0145
MCV, fL, median (IQR)	88.700 (85.850, 92.800)	91.700 (87.800, 95.650)	0.9999	0.9449–1.052	0.9968
AST/ALT, median (IQR)	0.630 (0.500, 0.848)	0.750 (0.600, 1.140)	0.4298	0.1920–0.8947	0.0307
Hypoproteinemia, n (%)	93 (88.571)	64 (75.294)	3.123	1.296–7.905	0.0129
GLB, g/L, median (IQR)	23.710 (20.230, 27.470)	28.030 (23.050, 30.880)	0.9209	0.8643–0.9784	0.0088

### 3.4 Outcomes

This study revealed that the 30-day mortality rate among acute leukemia patients with bloodstream infections was 10.5% (20/190), with a mortality rate of 9.5% (10/105) in multidrug-resistant bacterial infections and 11.8% (10/85) in non-multidrug-resistant bacterial infections. No significant difference was observed between the two groups (*P* = 0.6168), as shown in Table 1.

**FIGURE 3:**
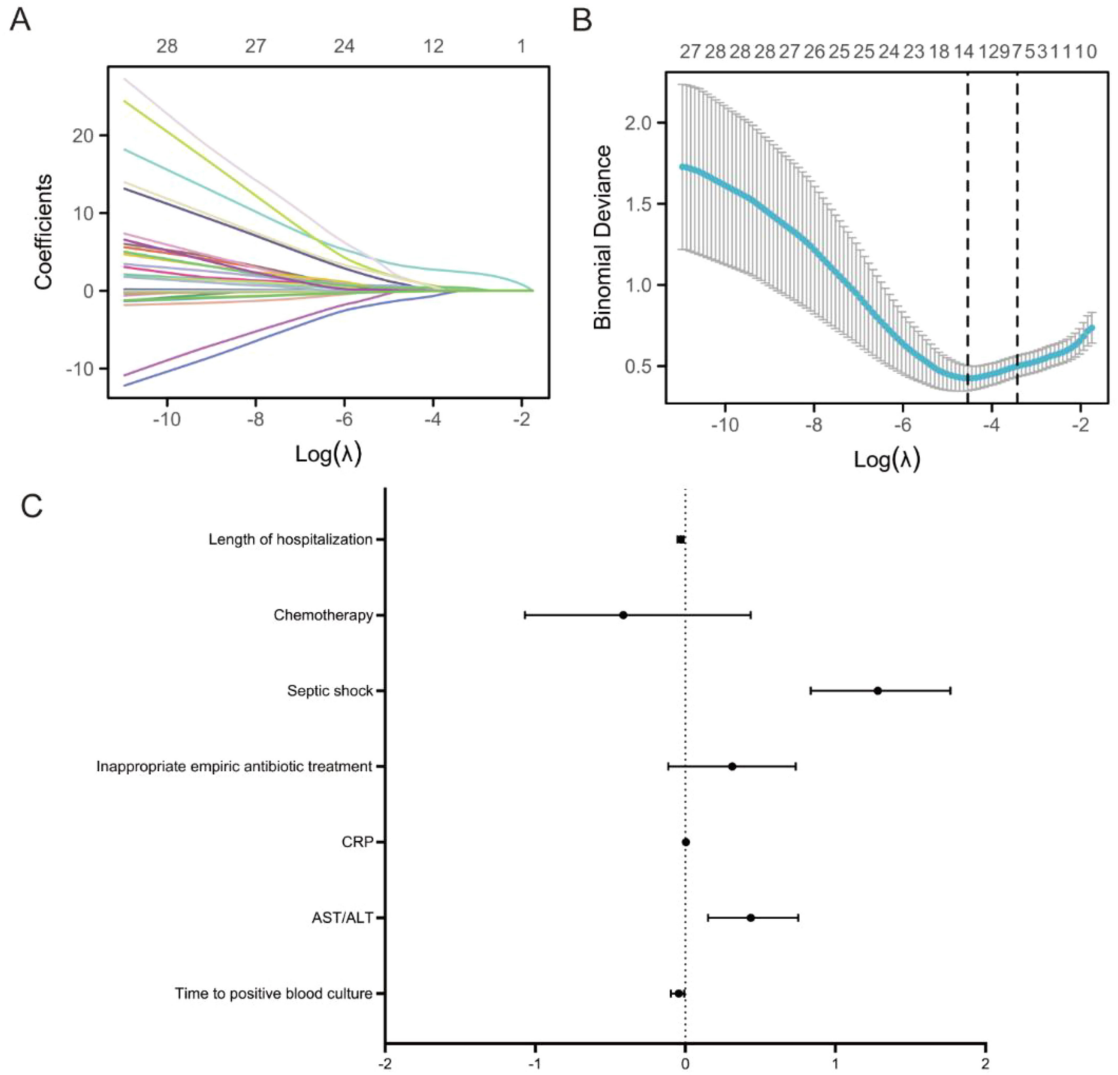
Risk factors for 30-day mortality after bloodstream infection in adult patients with acute leukemia screened by Lasso regression, and the ROC curve of the corresponding multivariate logistic regression model. **(A)** Plot of the LASSO coefficients. The horizontal axis represents the logarithmic value of lambda [log(λ)], and the vertical axis represents the coefficient values of the variables. **(B)** Cross-validation error curve. Bottom *x*-axis: represents the logarithm of lambda [log(λ)]. Numbers on the top *x*-axis: represent the number of non-zero coefficient variables corresponding to each lambda value. *y*-axis: represents the model’s -2 times the log-likelihood function value (deviance) under different metrics. **(C)** Forest plot of univariate logistic regression analysis for factors associated with 30-day mortality.

Through LASSO regression, it was identified that length of hospitalization, Chemotherapy, Septic shock, Inappropriate empiric antibiotic treatment, CRP, AST/ALT and Time to positive blood culture might be potential risk factors for bloodstream infection-related mortality in adult patients with acute leukemia. Considering the small sample size for 30-day mortality, we followed by univariate logistic regression analysis on the selected variables, to preliminarily evaluate factors associated with 30-day mortality risk in adult patients with acute leukemia. We can find that Septic shock (OR = 19.19, 95% CI: 6.849–58.47, *p* < 0.0001), CRP (OR = 1.010, 95% CI: 1.004–1.016, *p* = 0.0007), and the AST/ALT ratio (OR = 2.732, 95% CI: 1.417–5.643, *p* = 0.0063) may be associated with an increased risk of 30-day mortality, whereas Length of hospitalization (OR = 0.9312, 95% CI: 0.8863–0.9725, *p* = 0.0025) may serve as a protective factor, as detailed in Figure 3 and Table 5.

**TABLE 5:** Univariate analysis of 30-day mortality risk factors in adult acute leukemia patients with bloodstream infection.

Variable	Non-survivors (*n* = 20)	Survivors (*n* = 170)	OR	95% CI	*P*-value
Length of hospitalization, d, median (IQR)	16.500 (7.000, 23.500)	25.000 (17.000, 32.250)	0.9312	0.8863–0.9725	0.0025
Chemotherapy, n (%)	18 (90.000)	163 (95.882)	0.3865	0.08539–2.723	0.2574
Septic shock, n (%)	13 (65.000)	15 (8.824)	19.19	6.849–58.47	< 0.0001
Inappropriate empiric antibiotic treatment, n (%)	9 (45.000)	46 (27.059)	2.054	0.7687–5.431	0.1436
CRP, mg/L, median (IQR)	197.800 (78.170, 247.4)	68.780 (21.220, 147.700)	1.010	1.004–1.016	0.0007
AST/ALT, median (IQR)	1.035 (0.695, 1.658)	0.670 (0.520, 0.900)	2.732	1.417–5.643	0.0063
Time to positive blood culture, h, median (IQR)	10.350 (8.900, 12.530)	12.500 (10.150, 18.550)	0.9025	0.8002–0.9823	0.0531

## 4 Discussion

Antibiotic resistance is a global concern. Despite garnering significant attention from numerous researchers, clinical data and epidemiological studies on BSIs caused by antibiotic resistance in patients with acute leukemia are limited. This study included 190 adult patients with acute leukemia and BSIs, of whom 105 (55.263%) were infected with MDR bacteria, consistent with the findings of other studies (Wu M. et al., 2025). Owing to the limited availability of effective drugs for MDR bacterial infections, preventing the emergence of such pathogens remains a primary challenge.

In this study, we statistically analyzed the microbial distribution and resistance profiles of 105 MDR bacterial isolates. We found that Gram-negative bacteria accounted for a relatively large proportion (71.429%) and were more likely to be MDR, which is slightly higher than the rate reported in a study from a tertiary hospital in southwestern China (62.1%) (Wang et al., 2024). This dissimilarity may indicate the influence of geographical differences on the prevalence of MDR bacteria. Furthermore, the prevalence of MDR bacteria may vary geographically owing to different antibiotic use norms, prescription behaviors, and infection prevention strategies (Yehya et al., 2025; Beig et al., 2024; Lee et al., 2023). Among the 75 MDR Gram-negative bacteria isolated in this study, *E. coli* and *K. pneumoniae* constituted a significant proportion, concurring with the findings of several global studies (Zhao et al., 2020; Wang et al., 2024; van Leeuwen et al., 2025; Schonardie et al., 2023; Rejthar et al., 2025; Wu J. et al., 2025; Wu M. et al., 2025). Regarding antimicrobial susceptibility, nearly all MDR *E. coli* isolates exhibited strong resistance to fluoroquinolones, folate metabolism inhibitors such as trimethoprim/sulfamethoxazole, penicillins such as piperacillin, and most cephalosporins. MDR *K. pneumoniae* show relatively low susceptibility to fluoroquinolones and most cephalosporins.

Among the MDR Gram-positive cocci, *E. faecium* accounted for the greatest proportion, followed by *S. hominis*, which differs from previous findings (Rejthar et al., 2025). This suggests that geographical variations are more pronounced in Gram-positive cocci. MDR Gram-positive cocci exhibit strong resistance to both penicillin and erythromycin, whereas their susceptibility to fluoroquinolones is suboptimal.

Consistent with our study findings, a substantial number of studies have included invasive procedures as risk factors for MDR infections in patients with acute leukemia (Santoni et al., 2025; Averbuch et al., 2025; Martynov and Schoenberger, 2021). Invasive devices (such as PICC, CVC, and urinary catheters) are widely used in patients with acute leukemia. Disruption of the physical skin barrier significantly increases the risk of MDR bacterial infections.

In patients with acute leukemia, hypoproteinemia is a clinically significant common feature that acts as an independent risk factor for poor prognosis across different infection scenarios. Studies have shown that hypoproteinemia is an independent predictor of increased 30-day all-cause mortality in patients with hematological malignancies who develop Enterobacterales BSIs. Similarly, in patients with Pseudomonas infection, severe hypoproteinemia is independently associated with progression to septic shock (Cui et al., 2025; Jeddi et al., 2011; Garcia et al., 2013). Although malnutrition is widely recognized as a common risk factor for infections in immunocompromised hosts, direct evidence linking hypoproteinemia to multidrug-resistant bloodstream infections specifically in adult acute leukemia patients remains scarce and underexplored.

A limitation of this retrospective study is the inability to fully distinguish the temporal relationship between hypoproteinemia and MDR-BSI. Since serum total protein levels were measured within 48 h of BSI diagnosis, it is difficult to differentiate whether hypoproteinemia acted as a predisposing risk factor (reflecting pre-existing chronic malnutrition common in acute leukemia) or merely reflected the severity of the acute illness (such as systemic inflammation and capillary leakage). However, considering that patients with acute leukemia frequently suffer from chronic protein depletion due to the malignancy and prior chemotherapy, the observed association suggests that a chronic hypoproteinemic state likely establishes a high-risk internal environment for subsequent MDR infections. Therefore, regardless of the direction of causality, hypoproteinemia remains a valuable bedside marker for identifying high-risk patients who may benefit from enhanced nutritional support and closer monitoring. The precise causal relationship warrants further validation in prospective studies with serial protein measurements.

In the multivariate logistic regression analysis, elevated globulin levels were protective factors. Elevated globulin levels (OR = 0.9209, *p* = 0.0088) demonstrated protective effects. Globulins include immunoglobulins and various inflammation-related proteins; higher levels may reflect activated and sustained immune function, thereby enhancing host defense against MDR pathogens. Oral infection was significantly associated with a lower incidence of MDR-BSI (OR = 0.3450, *p* = 0.0145), which may be explained by the earlier initiation of antibiotic therapy or enhanced oral care in these patients, indirectly reducing MDR colonization and infection. this finding likely reflects detection bias and the timeliness of intervention. Oral lesions are readily visible and symptomatic, leading to heightened clinical vigilance and earlier initiation of antimicrobial therapy compared to patients with occult sources of infection. This early intervention may prevent the escalation of infection or the selection of resistant organisms. Therefore, oral infection should be viewed not as a protective factor, but as a visible clinical alarm that facilitates prompt and effective treatment. An elevated AST/ALT ratio was also significantly associated with a lower incidence of MDR-BSI (OR = 0.4298, *p* = 0.0307); a lower ratio (relatively higher ALT) is commonly observed in viral hepatitis and other hepatocellular injuries, which are often associated with more pronounced systemic inflammation and immune dysregulation and may increase susceptibility to MDR infection.

Collectively, our findings illustrate that MDR BSIs are the product of a multifaceted risk landscape. Directly actionable factors in clinical practice include minimizing invasive procedures and addressing the nutritional status, particularly hypoproteinemia. Other identified parameters, such as oral infection, AST/ALT ratio, and globulin levels, may potentially serve as integrative biomarkers reflecting the systemic susceptibility, immune competence, or specific management context of a patient, rather than being direct causative factors. This risk profile suggests that infection prevention in high-risk patients should involve a multifaceted strategy. Although certain factors demonstrated a protective association in our analysis, they should be interpreted as statistical links within this specific cohort and caution is warranted to avoid overinterpreting them as direct therapeutic targets or inferring causality. Future prospective studies are necessary to validate these risk associations and translate them into effective and precise preventive protocols. It is imperative to clarify that the associations identified between hypoproteinemia, elevated AST/ALT ratios, and MDR-BSI reflect statistical correlations rather than definitive causal relationships. Both hypoproteinemia and hepatic dysfunction are nonspecific markers of systemic illness in acute leukemia patients. They may be influenced by multiple confounders, including the underlying malignancy, the intensity of prior chemotherapy, sepsis-induced organ dysfunction, and baseline comorbidities. For example, an elevated AST/ALT ratio may indicate hepatic injury from chemotherapy or systemic inflammation, which in turn alters immune competence and antimicrobial pharmacokinetics, thereby creating an environment conducive to MDR emergence. Therefore, these variables should be regarded as integrated markers of physiological reserve and disease burden rather than isolated causative agents.

Among the 190 patients included in this study, 20 (10.5%) died within 30 days of infection, which is slightly lower than the rates reported in other studies (El Omri et al., 2024; Wu M. et al., 2025; Murali et al., 2016). MDR K. pneumoniae is associated with higher 30-day mortality than non-MDR K. pneumoniae (Wu J. et al., 2025), and multidrug resistance has been identified as a risk factor for mortality (Willems et al., 2023; Trecarichi et al., 2019; Cattaneo et al., 2018). In this study, we did not find any effect of MDR bacterial infection on mortality. This finding can be contextualized within the specific clinical environment of our institution. First, our microbiology laboratory supports a rapid diagnostic workflow that uses matrix-assisted laser desorption/ionization time-of-flight mass spectrometry, which enables prompt pathogen identification. Our hematology department adheres to a stringent protocol-based antimicrobial stewardship program and multidisciplinary consultation process (involving the Microbiology Laboratory, Infectious Diseases Department, and Pharmacy Department) when managing BSIs in patients with acute leukemia. We hypothesized that the combination of rapid microbiological guidance and proactive, protocol-driven therapeutic escalation may have attenuated the independent impact of MDR status on mortality, as effective therapy was not delayed. Nevertheless, the single-center study design and sample size limitations necessitate cautious interpretation of this result, and this hypothesis requires validation in larger multi-center studies.

We found that Septic shock, CRP, and the AST/ALT ratio may be associated with an increased risk of 30-day mortality. Septic shock has previously been reported as an independent risk factor for mortality at 30 days after BSI in patients with acute leukemia (Kessel et al., 2025; Magiorakos et al., 2012; Ma et al., 2024). Septic shock is often accompanied by multiple organ dysfunction, and patients exhibit poor compensatory capacity, which significantly increases mortality (Chang et al., 2025; Jaime-Pérez et al., 2024; Wang et al., 2025). Therefore, early identification and implementation of aggressive anti-infective treatment to prevent septic shock are imperative.

Laboratory indicators, such as the inflammatory marker CRP and measures of liver function, reflect a patient’s baseline organ function to some extent. Their impact on mortality outcomes may be mediated by their role in triggering or being associated with septic shock. This study was retrospective in nature, and a longitudinal prospective study may provide a clearer explanation of this phenomenon (Gradel et al., 2020). Nevertheless, timely anti-inflammatory and anti-infective treatments, along with appropriate correction of abnormal metabolic conditions related to liver function, are more conducive to reducing mortality risk in patients with acute leukemia. The association observed between shorter hospital stays and higher mortality is likely due to shorter overall hospital stays due to early death after BSI rather than the shorter length of hospital stay itself increasing the risk of death.

This study had several limitations. First, it was a single-center retrospective analysis, which may account for discrepancies in certain influencing factors compared to other studies. Second, the distribution of microbial flora is highly influenced by regional variations, and this study only included patients with acute leukemia from the northeast coastal region of China. Future multi-center, multi-regional studies are required. Furthermore, the sample size of patients with acute leukemia included in this study was insufficient, preventing us from drawing a conclusion regarding the association between MDR bacterial infections and mortality risk. Detailed data regarding specific chemotherapeutic regimens, treatment intensity, and phases were not collected in this retrospective study. Acute leukemia treatment protocols are often highly individualized and adjusted based on patient tolerance and response, making standardized categorization challenging. The lack of granular oncological treatment data limits our ability to analyze how specific drugs or intensities may interact with the risk of MDR-BSI. Future prospective studies should aim to integrate detailed chemotherapeutic data to address this gap.

Routine rectal swab screening for resistant pathogens has not yet been widely implemented in most Chinese hematology centers, including ours during the study period. Due to the lack of systematic baseline colonization data, we could not analyze the specific contribution of colonization pressure to MDR-BSI. This limitation highlights the importance of focusing on modifiable clinical indicators, such as hypoalbuminemia and invasive procedures, to predict infection risk in real-world clinical settings where universal screening is not feasible. Although the COVID-19 pandemic did not demonstrate a significant impact on MDR prevalence in the present study, its influence on clinical practice is undeniably complex. Validating these effects will require a longer study period and extended follow-up. In our future studies, we will also integrate the molecular genetic profiles of multidrug-resistant strains to explore the clinical characteristics of MDR infections from multiple dimensions.

## 5 Conclusion

This study analyzed BSIs in 190 adult patients with acute leukemia and revealed a high incidence of MDR bacterial infections, with a predominance of Gram-negative bacteria, particularly E. coli and K. pneumoniae, whose resistance profiles were consistent with global trends. Invasive procedures and hypoproteinemia as independent risk factors for MDR bacterial infections. Septic shock, CRP, and the AST/ALT ratio may be associated with an increased risk of 30-day mortality, although MDR bacterial infection itself was not found to directly increase mortality risk, this may be attributed to the sample size and single-center nature of the study, the limited number of mortality events resulted in insufficient statistical power to detect a true survival difference between the groups. This study contributes to the epidemiological data from northeastern coastal region of China and emphasizes the need to optimize infection prevention and treatment strategies based on local pathogen profiles. These findings may aid in the early identification of high-risk patients and inform clinical decision-making. However, the conclusions were limited by the retrospective single-center design and sample size of the study, underscoring the necessity of future multicenter prospective studies for further validation.

## Data Availability

The original contributions presented in this study are included in the article/Supplementary material, further inquiries can be directed to the corresponding author.

## Supplementary material

Supplementary Figure 1 Comparison of microbiological trends and MDR prevalence.
