# Supplementary material for: Clinical characteristics and risk factor analysis of multidrug-resistant bacterial bloodstream infections in adult acute leukemia patients
Source: Front Microbiol. 2026 Jun 4;17:1850927. doi: 10.3389/fmicb.2026.1850927 (PMC13275475; doi:10.3389/fmicb.2026.1850927)
Supplement: Supplementary file 1 [file Table_1.docx]

| Years | 2019 | 2020 | 2021 | 2022 | 2023 | 2024 | 2025 |
| --- | --- | --- | --- | --- | --- | --- | --- |
| MDR prevalence（%） | 56.67 | 69.23 | 57.14 | 66.67 | 56.00 | 35.14 | 54.17 |
| Number of positive blood cultures | 30 | 26 | 21 | 27 | 25 | 37 | 24 |
| Number of MDR isolates | 17 | 18 | 12 | 18 | 14 | 13 | 13 |

Supplementary Table S1. Microbiology and MDR prevalence statistics
