# Supplementary figures and images for: Clinical characteristics and risk factor analysis of multidrug-resistant bacterial bloodstream infections in adult acute leukemia patients
Source: Front Microbiol. 2026 Jun 4;17:1850927. doi: 10.3389/fmicb.2026.1850927 (PMC13275475; doi:10.3389/fmicb.2026.1850927)

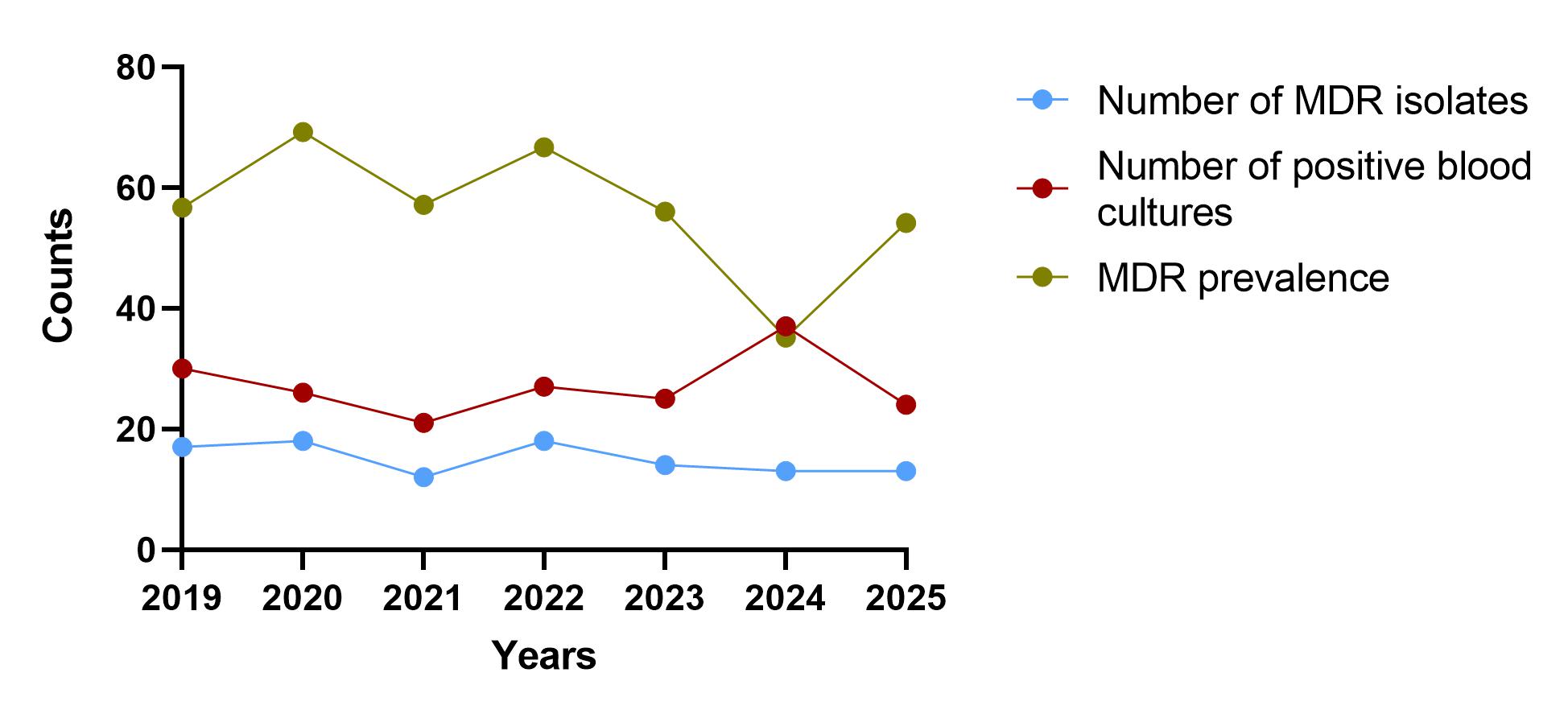

Supplement: Supplementary Figure 1 — Comparison of microbiological trends and MDR prevalence. [file Image_1.jpeg]
